# Challenges and opportunities for hydrogen production from microalgae

**DOI:** 10.1111/pbi.12516

**Published:** 2016-01-23

**Authors:** Melanie Oey, Anne Linda Sawyer, Ian Lawrence Ross, Ben Hankamer

**Affiliations:** ^1^ Institute for Molecular Bioscience The University of Queensland St Lucia Qld Australia; ^2^ Ruhr‐University Bochum Bochum Germany

**Keywords:** algae, solar, hydrogen, water, renewable energy, fuel

## Abstract

The global population is predicted to increase from ~7.3 billion to over 9 billion people by 2050. Together with rising economic growth, this is forecast to result in a 50% increase in fuel demand, which will have to be met while reducing carbon dioxide (CO_2_) emissions by 50–80% to maintain social, political, energy and climate security. This tension between rising fuel demand and the requirement for rapid global decarbonization highlights the need to fast‐track the coordinated development and deployment of efficient cost‐effective renewable technologies for the production of CO_2_ neutral energy. Currently, only 20% of global energy is provided as electricity, while 80% is provided as fuel. Hydrogen (H_2_) is the most advanced CO_2_‐free fuel and provides a ‘common’ energy currency as it can be produced via a range of renewable technologies, including photovoltaic (PV), wind, wave and biological systems such as microalgae, to power the next generation of H_2_ fuel cells. Microalgae production systems for carbon‐based fuel (oil and ethanol) are now at the demonstration scale. This review focuses on evaluating the potential of microalgal technologies for the commercial production of solar‐driven H_2_ from water. It summarizes key global technology drivers, the potential and theoretical limits of microalgal H_2_ production systems, emerging strategies to engineer next‐generation systems and how these fit into an evolving H_2_ economy.

## Introduction

The global economy is valued at ~US$100 Tn pa (CIA, 2014) and is powered by the $6 Tn pa energy sector (Dittrick and Izundu, 2008), with ~80% provided in the form of fuel and ~20% as electricity (IEA, 2009). While renewable electricity generation systems are being deployed rapidly, renewable fuel technologies are ~10–20 years behind on the development curve. This is due to the challenges of reducing fuel production costs towards ~$100 per barrel of oil equivalent to compete with fossil fuels, and the need to improve ‘Energy Return on Energy Invested’ (ERoEI – a measure of process efficiency) and reduce greenhouse gas (GHG) emissions.

Additionally, by 2050 the human population is forecast to increase from 7.3 billion to over 9 billion people (USCB, 2013), and together with continued global economic growth, this is projected to require 50% more fuel (IEA, 2010), 70% more food (FAO, 2009), 50% more fresh water (OECD, 2014) and cuts in CO_2_ emissions of 80% (IPCC, 2007; Stocker, 2013) to maintain political, social, fuel and climate security. It is therefore critical to fast‐track the development of ‘commercially‐ready’ CO_2_ neutral fuel systems that do not compete with food and water needs. The importance of this challenge is highlighted by the common vision of the Group of 7 (G7) nations to achieve GHG reductions of 70% by 2050 based on 2010 figures (G7, 2015).

## Solar fuel options

The production of clean fuels at a globally significant level requires a renewable energy source sufficiently large to drive this process. Solar energy is by far the largest energy source available to us, with 5500 ZJ (1 ZJ = 10^15^ J) (Smil, 2008) reaching the Earth's atmosphere every year. Of this, 1300 ZJ is photosynthetically active radiation (PAR) available at the Earth's surface (Oey *et al*., 2013). This annual irradiance level (1300 ZJ/year) is ~2300× the total global energy demand (0.56 ZJ/year) (BP, 2011) and dwarfs the combined energy from all reported oil, coal, gas and uranium reserves (Stephens *et al*., 2010b). Photosynthetic organisms have over 3 billion years, evolved intricate photosynthetic systems capable of tapping into this abundant solar resource for the production of food, fuel and atmospheric oxygen (O_2_) and not only support life on Earth, but provide a basis for next‐generation solar fuel production.

First and second generation crop‐based biofuels include sugar and corn ethanol, as well as lignocellulosic ethanol from woody biomass and agricultural residues. However, these technologies require arable land and fresh water, and so ultimately result in ‘*food vs fuel*’ or ‘*forest vs fuel*’ competition (e.g. palm oil). To achieve a transition from ‘*food/forest vs fuel*’ scenarios to a ‘*food and fuel*’ future, next‐generation biofuel systems will need to expand photosynthetic capacity into urban areas, onto nonarable land (~25% of the Earth's surface), or into the oceans to utilize alternative water sources and conserve arable land (~3% of the Earth's surface) (Stephens *et al*., 2012). Microalgae and cyanobacteria systems can be located on nonarable land (and potentially in urban areas and oceans), cultivated at least in part in saline and/or waste water, facilitating increased nutrient and water recycling, and achieve higher yields than crop plants due to the ability to optimize light distribution, CO_2_ supply and production conditions and thus offer advantages over crop‐based biofuel feed stocks (Pulz and Gross, 2004).

It can be calculated that at a 2% solar to biomass conversion efficiency, which is already achievable at demonstration scale for biomass production in microalgal systems, ~2.15% of the Earth's surface would be required to supply the current global energy demand in the form of biomass at average illumination levels (0.56 ZJ/year/1300 ZJ/year ×100/2 = 2.15%). Subsequent production of fuels (e.g. oil, bio‐diesel, ethanol, methane and H_2_) reduces this efficiency, but this is offset, at least in part, by the fact that natural photosynthesis can attain theoretical efficiencies of ~6% (Melis and Happe, 2001; Zhu *et al*., 2008, 2010). Engineered microalgae and artificial systems, particularly those capable of H_2_ production, can theoretically achieve higher efficiencies, but require further development (Blankenship *et al*., 2011; Kruse *et al*., 2005b; Melis, 2009). This article reviews advances in the development of microalgae‐based solar‐driven H_2_ production processes from water, with a particular focus on the challenges that must be overcome to deliver them and potential solutions that they offer.

## Microalgal H_2_ production

The increasing requirement for carbon emission reduction makes H_2_ more attractive as a fuel as its combustion yields only H_2_O. Furthermore, H2 has a 2.7–3.09 higher energy density (~120 (LHV) – 140 (HHV) kJ/g) than other hydrocarbon fuels (Gupta et al., 2013). Conventional, fossil fuel‐based H_2_ production methods (e.g. steam reformation of natural gas (50%), industrial oil and naphtha reforming (30%), coal gasification (18%) and fossil fuel driven water electrolysis (3.9%) (Gupta *et al*., 2013; Kalamaras and Efstathiou, 2013)) are costly, energy intensive and emit high levels of CO_2_. In comparison, renewable bio‐H_2_ production systems (such as microalgae and cyanobacteria systems) have the potential to be carbon negative and less energy intensive, as they operate at ambient temperature and pressure (Karthic and Joseph, 2012).

**Figure 1:**
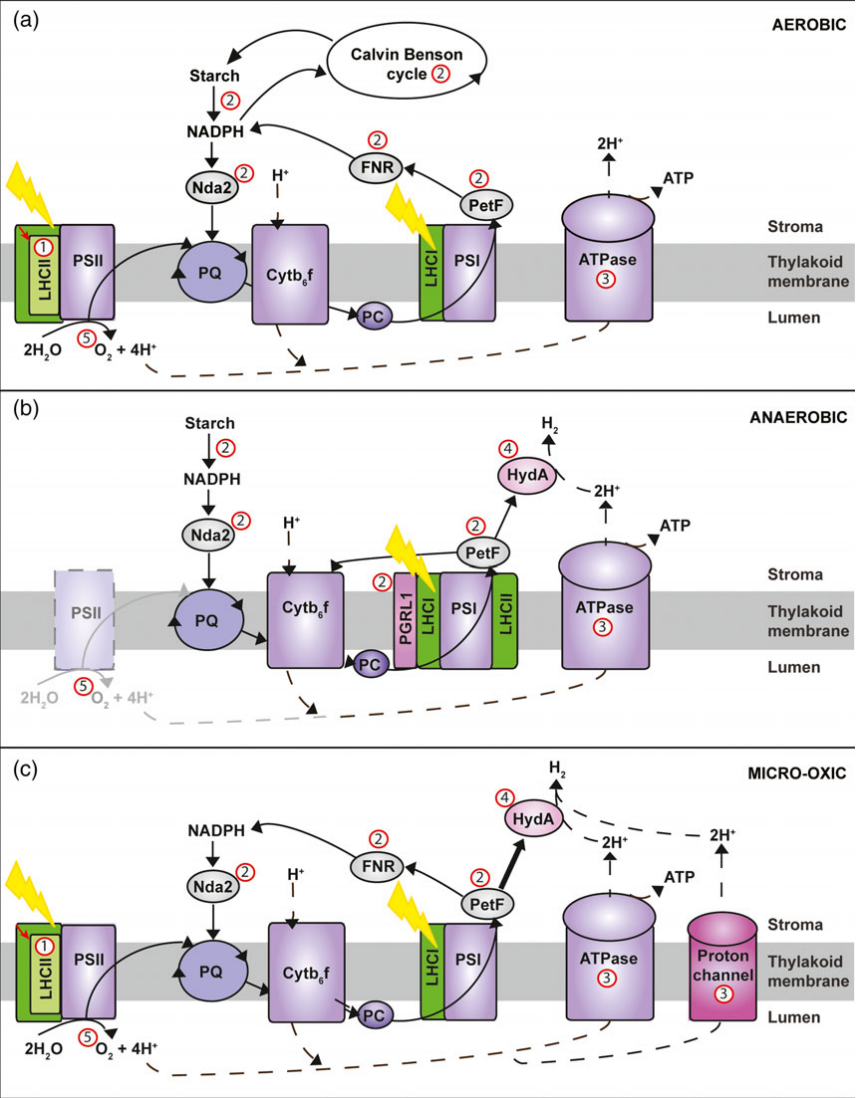
Overview of the photosynthetic microalgal H2 production from water. Aerobic (a) and anaerobic (b) stages of the two phase H2 production and Micro‐oxic continuous H2 production (c). Proton (H^+^) flow is marked with dashed lines, electron flow with continuous lines. Potential engineering targets for improved H_2_‐production are marked in red (labels #1**–**5) and described in detail in section ‘*Biological challenges and bottlenecks for microalgal H2‐production’*. *LHCII*: light‐harvesting antenna proteins of photosystem II; *PSII*: photosystem II reaction centre; *PQ*: plastoquinone pool; *Cytb*_*6*_ *f*: cytochrome b_6_f complex; *PC*: plastocyanin; *PGRL1*: proton gradient regulation like1 protein; *LHCI*: light‐harvesting antenna proteins of photosystem I; *HydA*: hydrogenase A; *PETF:* ferredoxin; *FNR*:* ferredoxin* (flavodoxin)‐NADP(H) reductase; *NDH*: NADPH‐dehydrogenase.

Hydrogen production using oxygen evolving photosynthesis (oxygenic photosynthesis) is the most advanced biological H_2_ production approach and the focus of this article. It uses solar energy to split water into protons (H^+^), electrons (e^−^) and O_2_ (Figure 1a) and recombines the derived H^+^ and e^−^ by either hydrogenase (Figure 1b) or nitrogenase enzymes to produce H_2_. The utilization of H^+^ and e^−^ derived from water photolysis for H_2_ production has only been reported in green algae and cyanobacteria, and provides the basis for the development of bio‐inspired artificial photosynthetic systems (Blankenship *et al*., 2011). The water photolysis process can be divided into indirect and direct pathways.

### Indirect water photolysis H_2_ production process

The indirect process can take place in both microalgae and cyanobacteria (Kruse *et al*., 2005a; Mathews and Wang, 2009; Melis and Happe, 2001; Melis *et al*., 2000; Rathore and Singh, 2013). Solar energy is first converted into chemical energy in the form of carbohydrates, which are then used as substrates for H_2_ production. Cyanobacteria, depending on the species, utilize both nitrogenases and hydrogenases for H_2_ production (Dutta *et al*., 2005), whereas microalgae rely solely on hydrogenases (Oncel *et al*., 2015). Nitrogenases have the advantage that they act unidirectionally, whereas hydrogenases are bidirectional (Melnicki *et al*., 2012). However, the high O_2_ sensitivity of both enzymes requires the separation of H_2_ evolution and CO_2_ fixation. In cyanobacteria, the reactions are either temporally separated during light and dark periods, or physically separated, with H_2_ production occurring in heterocysts and CO_2_ fixation in the vegetative cells (Dutta *et al*., 2005). The low ratio of heterocysts to vegetative cells (about 1:10) limits H_2_ production levels (Bandyopadhyay *et al*., 2010). High H_2_ production rates have been reported using cyanobacteria as catalysts (i.e. through the inhibition of cell replication) (Bandyopadhyay *et al*., 2010; Melnicki *et al*., 2012). However, the energy requirement for the nitrogenase reaction (4 ATP per H_2_) reduces H_2_ production and conversion efficiencies to less than half of the efficiencies expected from hydrogenase‐based indirect photolysis (LaurentPilon and Halil, 2014). In algal systems, the most common, but not ideal practice, is the temporal separation of the O_2_ and H_2_ production phase (aerobic and anaerobic phase, Figure 1) to circumvent oxygenic inactivation of the hydrogenase (further described below) (Kruse *et al*., 2005a; Melis and Happe, 2001; Melis *et al*., 2000).

### Direct water photolysis H_2_ production process

Direct photolysis has only been reported in microalgae, and involves the funnelling of e^−^ derived from the light‐driven water splitting reaction of photosystem II (Figure 1c) directly to a H_2_‐producing hydrogenase (Melis and Happe, 2001; Melis *et al*., 2000). While direct solar‐driven H_2_ production from water offers the highest theoretical photon conversion efficiency, achieving this is technically challenging due to the high O_2_ sensitivity of the hydrogenase. It requires customized cell lines and production systems which allow the simultaneous production of H_2_ and O_2_ as this co‐production is tightly down‐regulated in native strains (Melis *et al*., 2000).

To date, the highest bio‐H_2_ production efficiencies have been reported for microalgae (Scoma *et al*., 2012; Volgusheva *et al*., 2013). In microalgae, the first step of photosynthesis is the capture of solar energy by the light‐harvesting complex (LHC) proteins (Figure 1a). The LHC proteins are classified as LHCI or LHCII types depending on their predominant interaction with either photosystem I (PSI) or II (PSII), respectively. The LHC proteins belong to a large gene family, which in the green alga, *Chlamydomonas reinhardtii,* consists of over 20 members (Dittami *et al*., 2010). The excitation energy transferred to PSII by LHCII (Figure 1a) drives the photosynthetic water splitting reaction which converts H_2_O into H^+^, e^−^ and O_2_ (Eq. (1)).

The photons captured by LHCI and LHCII excite e^−^ derived from H_2_O, and drive their transfer along the photosynthetic electron transport chain (e^−^ flow is indicated by solid black lines in Figure 1a) from the PSII‐LHCII supercomplex via plastoquinone (*PQ*), cytochrome b_6_f (*Cyt)*, the PSI‐LHCI supercomplex and ferredoxin (*Fd*) (via the ferredoxin‐NADP^+^ oxido‐reductase; FNR) to finally reduce NADP^+^ to NADPH. NADPH, together with ATP, is used in the Calvin–Benson cycle and subsequent biochemical pathways to fix (i.e. reduce) CO_2_ to the sugars, starch, oils and other bio‐molecules which collectively form biomass.

Simultaneously, H^+^ are released into the thylakoid lumen by PSII and the PQ/PQH_2_ cycle (H^+^ flow is indicated by black dashed lines in Figure 1). These H^+^ generate an electrochemical H^+^ gradient across the thylakoid membrane, which drives ATP production via the flow of H^+^ back to the stroma through the ATP synthase.

Under normal aerobic conditions (Figure 1a), photosynthesis produces the carbohydrates that fuel mitochondrial respiration and cell growth. However, under anaerobic light conditions (Figure 1b), mitochondrial oxidative phosphorylation is largely inhibited by the lack of oxygen, leading to chloroplast over‐reduction and a slowing of the electron transport chain, which can ultimately result in photo‐damage and reduced ATP generation. Under these conditions, the H^+^ and e^−^ extracted from water by the remaining PSII or starch can be fed to the hydrogenase (HYDA) via the electron transport chain, which recombines the H^+^ and e^−^ to produce H_2_ (Eq. (2)).

Overall therefore, Eqs (1) and (2) describe solar‐driven H_2_ production from H_2_O. The hydrogenase essentially acts as a H^+^/e^−^ release valve by recombining H^+^ (from the medium) and e^−^ (from reduced ferredoxin) to produce H_2_ gas that is excreted from the cell.

(1) $$2H_{2}O\to 4H^{+}+4e^{-}+O_{2}$$

and

(2) $$4H^{+}+4e^{-}\to 2H_{2}$$

The high H_2_ production efficiency of microalgae is partly due to the high efficiency of the algal [FeFe]‐hydrogenase, which is 100‐fold higher than that of other hydrogenases (turnover rate of up to 10^4^ H_2_ molecules/s) (Lubitz *et al*., 2014; Volgusheva *et al*., 2013, 2015). The overall process is also attractive for sustainable applications as it occurs at ambient temperature and pressure. The volatility of H_2_ has the advantage that it prevents feedback inhibition and its separation into the gas phase assists harvesting, although there are technical challenges in terms of H_2_ containment and its separation from contaminating oxygen.

*Chlamydomonas reinhardtii* is one of the best studied microalgae species with respect to H_2_ production processes, availability of genetic tools and structural biology knowledge of the photosynthetic machinery (Nield *et al*., 2004; Posten and Walter, 2012; Tokutsu *et al*., 2012). It is not only able to produce H_2_ via oxygenic photolysis (direct and indirect), but also via dark fermentation (see below), in a process similar to that of other micro‐organisms (Eroglu and Melis, 2011; Stripp and Happe, 2009; Tolleter *et al*., 2011; Volgusheva *et al*., 2013; Wecker and Ghirardi, 2014). Of these pathways, light‐dependent H_2_ production is the most direct photon‐to‐fuel conversion process. However, the natural hydrogenase is only expressed and active under micro‐oxic or anaerobic conditions (Ghirardi *et al*., 1997) and is rapidly inactivated by the gradual accumulation of photosynthetically produced O_2_. While this provides the basis for a ~95% pure H_2_ stream (Kruse *et al*., 2005a) and potentially protects against the formation of explosive O_2_/H_2_ mixtures, under natural conditions it only allows for transient H_2_ production.

Currently, most algal systems are based on a simple process of temporal separation to circumvent the oxygenic inactivation of the hydrogenase (Melis *et al*., 2000). This involves first growing algae aerobically for biomass accumulation, ideally by CO_2_ fixation, during which oxygen is released and the reducing power stored, typically as carbohydrates (Figure 1a). This step is followed by sulphur (S) starvation. Among other changes, S deprivation reduces the rate of repair of a PSII reaction centre protein (D1), which contains the S‐containing amino acid methionine. Photo‐damage then reduces PSII to ~ 25% of the original level (Volgusheva *et al*., 2013). This drops the O_2_ production below the rate of mitochondrial respiration and achieves the anaerobic/micro‐oxic conditions which induce the expression of an active hydrogenase (Figure 1b). While anaerobiosis initially blocks the e^−^ transport from the remaining PSII in the phase preceding H_2_ production, the block is subsequently released and direct e^−^ transport to the hydrogenase occurs, with ~80% of the H_2_ being derived directly from PSII activity (Volgusheva *et al*., 2013). Under these conditions, the reactions described in Eqs (1) and (2) are effectively coupled, resulting in the overall process described in Eq. (3). However, they also limit the overall efficiency of the process.

To overcome the limitations of S deprivation, other approaches aim to develop O_2_‐tolerant hydrogenases or balance O_2_ production with metabolic O_2_ utilization (Oey *et al*., 2013). A further approach is the use of S microdosing (Figure 1c) (Kosourov *et al*., 2005) to allow PSII repair, the production of H^+^ and e^−^ from water and to balance O_2_ evolution with O_2_ utilization through mitochondrial respiration (Kruse *et al*., 2005a). This micro‐oxic approach has the advantage of being more efficient and can provide a basis for a continuous H_2_ production process. Another, recently published strategy is magnesium deprivation, which results in a decreased photosynthetic activity but increased respiration and starch accumulation allowing a prolonged H_2_ production period (>7 days). Under these conditions, functional PSII, which appears to be crucial for H_2_ production, was only reduced by 20%, implying a higher e^−^ availability to the hydrogenase (Volgusheva *et al*., 2015). Furthermore, the co‐cultivation of algae and bacteria has been presented as a new approach to achieve algal anaerobiosis via increased bacterial respiration (Lakatos *et al*., 2014). It is also of note that two algae strains are reported to be able to produce low levels of H_2_ under aerobic conditions (Hwang *et al*., 2014).

(3) $$\begin{array}{cc} 2H_{2}O\to & 2H_{2(\text{gas})}+O_{2(\text{extracted}\;\text{by}\;\text{respiration})}(\text{Typically}\;H_{2}\;\text{gas is}\;\\ & ∼\;95\%\;\text{pure}(\text{Kruse}\;\text{et al.},2005a)) \end{array}$$

## Biological challenges and bottlenecks for microalgal H_2_ production

In addition to *Chlamydomonas*, several other algal species, including species of *Chlorella* (Rashid *et al*., 2011), *Scenedesmus* (Schulz *et al*., 1998) and *Tetraselmis* (D'Adamo *et al*., 2014), have also been reported to produce H_2_ but at lower levels. The *C. reinhardtii* system has the advantage that all three genomes (nuclear, chloroplast and mitochondrial) have been sequenced and transformed, and much more detailed information is available on the organization of photosynthetic complexes during H_2_ production (Hemschemeier and Happe, 2011; Tolleter *et al*., 2011). Transformation techniques are well established and include particle bombardment (Debuchy *et al*., 1989; Kindle *et al*., 1989; Mayfield and Kindle, 1990), glass bead or silicon‐carbide whisker methods (Dunahay, 1993; Kindle, 1990), electroporation (Shimogawara *et al*., 1998) and *Agrobacterium tumefaciens*‐mediated gene transfer (Kumar *et al*., 2004). *Chlamydomonas* therefore remains the best characterized model organism for microalgal H_2_ production, associated genetic engineering and the identification of molecular bottlenecks.

### Optimizing light capture efficiency

The LHC antennae systems have a dual role: to capture photons and dissipate excess light energy to provide photoprotection (Niyogi, 1999; Pascal *et al*., 2005; Takahashi *et al*., 2006). Biomass production efficiency, at least in the laboratory, can be improved through LHC antenna reduction (Figure 1 – label #1), as this enhances light distribution through the bioreactors, enables the use of increased operational cell concentrations and can yield improved overall photosynthetic efficiencies of these systems (Beckmann *et al*., 2009; Melis *et al*., 1999; Mussgnug *et al*., 2007; Oey *et al*., 2013; Polle *et al*., 2003). Furthermore, *C. reinhardtii* antenna mutants have also been reported to exhibit an earlier onset of H_2_ production under S deprivation conditions (Oey *et al*., 2013), which is likely due to three factors: (i) improved light distribution leading to higher photon conversion efficiencies of the overall culture, (ii) the ability to lower the dissolved O_2_ concentration through the use of higher cell densities, which balance O_2_ production with metabolic load, and (iii) altered photo‐inhibition and stabilization of PSII required for subsequent H_2_ production (Volgusheva *et al*., 2013). Collectively these properties resulted in cultures exhibiting higher H_2_ production rates under the conditions tested (Oey *et al*., 2013).

The precise engineering of the LHC antenna requires a detailed understanding of the structural complexity and dynamic response of algae to light in larger scale production systems (de Mooij *et al*., 2015). To date, the engineering of the nuclear encoded antenna genes has utilized chemical or random insertion mutagenesis (Polle *et al*., 2002, 2003), the manipulation of antenna regulation proteins (e.g. NAB1 (Beckmann *et al*., 2009)) or *RNAi* knock‐down approaches (Mussgnug *et al*., 2007; Oey *et al*., 2013).

The least specific method of engineering antenna mutants involves the introduction of foreign DNA into the nuclear genome via *random insertion* (Zhang *et al*., 2014). As clear phenotypes are needed for the rapid screening of mutants, the expected light green phenotypes of putative antenna mutants seems an attractive selection criteria. However, while this approach identifies mutants impaired in antenna or chlorophyll synthesis, it does not select for specific multiple mutations likely required for optimal H_2_ production.

A more targeted approach to engineer antenna cell lines is via the indirect route of manipulating antenna regulation proteins. An example of this is the over‐expression of the translational repressor NAB1, which results in the specific down‐regulation of specific LHC proteins (Beckmann *et al*., 2009). This approach relies on cellular regulatory mechanisms and is therefore limited in its target scope. Another approach is *RNAi* mediated knock‐down (De Riso *et al*., 2009; Moellering and Benning, 2010; Molnar *et al*., 2007, 2009; Rohr *et al*., 2004; Schmollinger *et al*., 2010; Sun *et al*., 2008; Zhao *et al*., 2007, 2009) of specific target genes such as *LHC* genes, or genes involved in chlorophyll biosynthesis. A challenge for precise antenna engineering is that the coding regions of the *LHC* genes are highly homologous (Natali and Croce, 2015), which complicates the specific down‐regulation of target *LHC* genes. Additionally, the need to maintain the *RNAi* expressing mutants, typically through ongoing selective pressure, makes this approach less than ideal for industrial scale up. *RNAi* is therefore best suited for proof‐of‐principle studies to identify target genes.

The most elegant technologies facilitate precise and permanent genome editing enabling the fine tuning of antenna genes and adjustments to the pigment content, expanding the available solar spectrum (Blankenship and Chen, 2013; Chen and Blankenship, 2011). Several genome editing systems have been developed in recent years, including zinc finger nucleases (ZFN) (Sizova *et al*., 2013), transcription activator‐like effector nucleases (TALENs) (Gao *et al*., 2014) and the CRISPR/Cas systems (Cho *et al*., 2013; Mali *et al*., 2013), all of which rely on nuclease‐induced DNA strand breaks and endogenous cell repair mechanisms to obtain mutants with specifically edited genomes. This allows processes such as photosynthesis to be fine‐tuned for biotechnological applications. Another attraction of these techniques is the potential to develop mutants which are nongenetically modified organisms due to the transient introduction of the required nucleases. This would be a significant advance for large‐scale applications such as biofuel production, particularly given current legislative limitations on the use of GMO strains.

While ZFN (Sizova *et al*., 2013) and TALENs (Gao *et al*., 2014) have been used for genome editing in *Chlamydomonas,* they still involve labour‐intensive cloning steps. In contrast, the CRISPR/Cas system (Cho *et al*., 2013; Mali *et al*., 2013) promises to be a simpler technique only requiring the expression of an RNA guided nuclease, Cas9, to introduce strand breaks. The template for the guide RNA is fairly short (~23 bp), assisting with the targeted engineering of specific antenna genes given their high level of homology. Although successful CRISPR/Cas usage in *Chlamydomonas* is yet to be reported, it is anticipated that this technique will be possible in *Chlamydomonas* in the near future (Jiang *et al*., 2014), having already been successfully demonstrated in mammalian cells (Cong *et al*., 2013; Mali *et al*., 2013; Park *et al*., 2014), plants (Fauser *et al*., 2014; Feng *et al*., 2013; Xie and Yang, 2013) and zebra fish (Hwang *et al*., 2013). A patent application for its usage in the algal genus *Nannochloropsis* is also encouraging (Patent US 20140220638 A1).

### Electron supply to the hydrogenase and availability of reduced PETF

Limited electron flow to the hydrogenase is another potential bottleneck for sustainable H_2_ production (Hallenbeck and Benemann, 2002) (Figure 1 – label #2). This can occur due to the limited availability of reduced ferredoxin (PETF) as a result of other competing pathways (Winkler *et al*., 2011) (e.g. ferredoxin‐NADP^+^ reductase (FNR), sulphite reductase, nitrate reductase, glutamate synthase and fatty acid desaturases (reviewed in Hemschemeier and Happe, 2011)). The hydrogenase (HYDA) can accept e^−^ via a direct (PSII‐dependent; 2 photons per electron to HYDA) or indirect (PSII‐independent from starch; 3 photons per electron to HYDA) route (Chochois *et al*., 2009; Fouchard *et al*., 2005). To improve electron flow, PETF, FNR and the hydrogenase have all been engineered (Long *et al*., 2009; Lubner *et al*., 2011; Rumpel *et al*., 2014; Sun *et al*., 2013; Wittenberg *et al*., 2013; Yacoby *et al*., 2011), with a particular focus on the improvement of the affinity of the hydrogenase to PETF, the reduction of the affinity of PETF for FNR and the fusion of PETF and PSI with the hydrogenase. However, the identified candidates have so far only been tested *in vitro* and are yet to be assessed for their performance *in vivo*. Photosynthetic, H_2_ producing algal cells supply an excellent scaffold to carry out such proof of principle studies. While most engineering efforts have focused on the chloroplast‐localized nuclear encoded genes, recent efforts have been made towards the *in situ* overexpression of the hydrogenase in the plastid to uncouple it from its native control system (Reifschneider‐Wegner *et al*., 2014). Engineering has also focused on indirect targets, including electron competitors such as RuBisCo (Pinto *et al*., 2013), cyclic electron flow (Kruse *et al*., 2005a; Tolleter *et al*., 2011), starch degradation (Chochois *et al*., 2009) and respiration (Ruehle *et al*., 2008), with mutants in all of these pathways reportedly yielding increased H_2_ production levels. Finally, additional components have been added to the electron transfer pathway, including a plastid‐expressed NAD(P)H dehydrogenase (Baltz *et al*., 2014), a hexose transporter (Doebbe *et al*., 2007) and exogenous hydrogenases (Chien *et al*., 2012).

### Alteration of the thylakoid proton gradient

Proton supply to the hydrogenase is another potential bottleneck (Figure 1 – label #3). The transport of e^−^ from water to PETF via the photosynthetic electron transport chain is involved in establishing a proton gradient across the thylakoid membrane which drives ATP synthesis. While ATP production is essential during CO_2_ fixation, the ATP requirement drops during H_2_ production (Das *et al*., 2014) and electron transport is reduced at the point of *Cytb*_*6*_ *f* (Antal *et al*., 2009; Burgess *et al*., 2011), leading to an impaired dissipation of the proton gradient and therefore decreased proton availability for the hydrogenase and reduced H_2_ production. One strategy to improve H_2_ production is to artificially dissipate the proton gradient to increase H_2_ production transiently in the presence of the chemical uncoupler carbonyl cyanide *m*‐chlorophenyl hydrazine (CCCP), which causes an efflux of H^+^ from the thylakoid lumen into the stroma (Kruse *et al*., 2005a; Lee, 2013; Lee and Greenbaum, 2003). This suggests that the integration of a proton channel into the thylakoid membrane could more permanently restore proton and electron flow to the hydrogenase. Such a proton channel, however, would need to be inducibly expressed, as the addition of the uncoupler prior to anaerobiosis was found to abolish hydrogenase activity, suggesting that the proton gradient is important for initial hydrogenase expression (Lee, 2013) and aerobic growth. A similar strategy involves the development of a leaky ATPase to increase proton flow and reduce ATP production (Das *et al*., 2014; Robertson *et al*., 1990). Reduced ATP production caused by the introduction of a proton channel or mutated ATPase may additionally reduce reactions competing for reducing equivalents and therefore increase electron supply to the hydrogenase (Kumar and Das, 2013).

### Oxygen sensitivity of the hydrogenase

Sustained H_2_ production under standard growth conditions remains a major challenge, as O_2_ sensitivity of the hydrogenase is a multifaceted problem. This is because O_2_ oxygen inhibits not just hydrogenase enzyme function, but also transcription and protein maturation (Cohen *et al*., 2005). Interestingly, two algae strains were recently reported to be able to express the hydrogenase in the presence of more than 21% O_2_ and to produce low levels of H_2_ at 15% atmospheric O_2_ (Hwang *et al*., 2014). Several genetic engineering approaches have also been tested to reduce the O_2_ sensitivity of the hydrogenase (Figure 1 – label #4), including random mutagenesis (Flynn *et al*., 2002) and targeted mutagenesis of the catalytic site to restrict O_2_ access (Stiebritz and Reiher, 2012). While engineering approaches have been successful for a bacterial [NiFe] hydrogenase (Dementin *et al*., 2009), this has so far not been the case with the microalgal [FeFe] hydrogenase. Despite this, the identification of hydrogenases with reduced O_2_ sensitivities will increase the scope for genetic engineering. A hydrogenase with a reduced O_2_ sensitivity would open up a direct path to H_2_ production from oxygenic water splitting, but would also lead to the co‐production of O_2_, which then requires subsequent gas separation. However, this approach would overcome the need for a two phase aerobic/anaerobic process, potentially enabling a continuous H_2_ production process and eliminating the need for ATP and NADPH, which are required to produce starch‐ and oil‐based feed stocks for alternative fuels. Achieving this in thermophiles would be expected to further improve reaction kinetics.

### Oxygen evolution and removal

A different approach used to alter O_2_ sensitivity and extend H_2_ production is to control the O_2_ concentration of the culture. Increasing the cell density and respiratory load can result in an earlier onset of anaerobiosis and H_2_ production (Oey *et al*., 2013; Schönfeld *et al*., 2004). Furthermore, the down‐regulation of PSII (Surzycki *et al*., 2007) and engineering of O_2_ evolution activity (Makarova *et al*., 2007; Scoma *et al*., 2012; Torzillo *et al*., 2009) have been exploited (Figure 1 – label #5). Leghemoglobins, which are able to sequester O_2_, have also been expressed in *C. reinhardtii* (Wu *et al*., 2010, 2011), and in a different approach, a sulphate permease mutant was developed, which allowed greater control of S deprivation (Chen *et al*., 2005).

### H_2_‐production improvement strategies ‐ Supportive targets

Despite the obvious benefit of manipulating targets directly involved in H_2_ production, engineering more indirect targets to simplify cultivation, analysis and H_2_ production is also important. The ability to easily report lumen pH (Benčina, 2013; Demaurex, 2002), hydrogenase expression and key metabolic pathways such as sulphur, starch and lipid metabolism, combined with external control of gene expression levels and physiological mechanisms via the supply of stimuli such as hormones or light, would enable the rapid dissection of the changes in cell state occurring during H_2_ production. New genome editing tools (Cong *et al*., 2013; Fauser *et al*., 2014; Feng *et al*., 2013; Hwang *et al*., 2013; Mali *et al*., 2013; Park *et al*., 2014; Xie and Yang, 2013) will provide scope for sophisticated gene regulation and diagnosis similar to those used in mammalian systems including inducible promoters and self‐altering genes (Cre‐lox (floxed) systems) (Hajdukiewicz *et al*., 2001; Oey *et al*., 2009), highly specific reporter systems and further tools for improving research and industrial use.

### High‐throughput screening methods

The development of new H_2_ screening methods can also accelerate the engineering of enhanced H_2_‐producing algal strains. To date, H_2_ mutants have been detected through a chemo‐chromic sensing assay, which utilizes a palladium/tungsten oxide film (Seibert *et al*., 1998) or in screens specific to the created mutants (Kruse *et al*., 2005a; Ruehle *et al*., 2008; Stapleton and Swartz, 2010; Tolleter *et al*., 2011). A recent H_2_‐detection screen developed (Wecker and Ghirardi, 2014), which uses *Rhodobacter capsulatus* expressing an emerald green fluorescent protein (emGFP) driven by the *hupR* H_2_‐sensing promoter as a reporter for H_2_ production, offers a simple, inexpensive and semi‐quantitative screening method. The development of a chlorophyll fluorescence assay (Godaux *et al*., 2013) and an inorganic hydrogenase active site mimic will also allow for quicker screening of hydrogenase mutants (Berggren *et al*., 2013; Esselborn *et al*., 2013).

## Microalgae: a blueprint and evaluation platform for bio‐inspired hydrogen production

Natural microalgae‐based light‐driven H_2_ production processes serve as a blueprint and testing platform for the development of bio‐inspired artificial solar H_2_ production systems. For example, at the level of component development, the atomic structure of the light‐harvesting antenna systems provides valuable insights into the design of engineered and artificial light capturing pigments and systems (Qin *et al*., 2015). Another example is the design of new water splitting catalysts based on the manganese cluster of PSII (Brimblecombe *et al*., 2010; Tsui *et al*., 2013; Zhang *et al*., 2015). The arrangement of the redox‐active components of the photosystems has also been studied extensively, and other research has led to the development of self‐assembling synthetic scaffolds able to optimize the coordination and spacing of the components of artificial photosynthetic systems (Farid *et al*., 2013; Mancini *et al*., 2013; Weingarten *et al*., 2014). In this context, it is notable that the hydrogenase of *C. reinhardtii* can be prepared in large quantities as an apo‐protein and be readily reconstituted with its iron sulphur clusters to form an active hydrogenase (Esselborn *et al*., 2013). Through the use of such components, it is therefore theoretically possible to build light‐driven redox‐active systems which split water and use the H^+^ and e^−^ for H_2_ production. However, the economic viability and ERoEI of both algae and bio‐inspired artificial systems will have to be determined later in the development cycle.

## Challenges of scale up

### Coordinated biological engineering

The selection and engineering of high‐efficiency microalgae cell lines is central to this process. Lessons learnt from the optimization of microalgal H_2_ production suggest that the highest efficiency strains will require not single mutations, but significant re‐engineering of the H_2_ production process. For example, down‐regulating the light‐harvesting proteins has benefits as light green cell lines enable higher cell densities to be used, resulting in more rapid O_2_ scavenging, the early onset of H_2_ production and an overall higher H_2_ yield (Oey *et al*., 2013). The yield of H_2_ can also be increased by locking the photosynthetic electron transport chain into the State 2 configuration and by reducing cyclic electron transport, which competes with the hydrogenase for e^−^ (Kruse *et al*., 2005a; Tolleter *et al*., 2011). Additionally, lessening the competition between the hydrogenase and FNR for electrons for example, by re‐engineering the electron donors of the hydrogenase could further improve H_2_ production (Rumpel *et al*., 2014). The ultimate goal would be the combination of all of these targeted mutations into a single production strain for continuous H_2_ production. It would essentially allow the microalgae cells to act as catalysts under micro‐oxic conditions where O_2_ production and consumption are in balance (Figure 1c), and the water splitting reaction is active at the same time as the hydrogenase. This would enable a 2 photon vs 3 photon electron transfer steps from water to H_2_, which represents a 33% improvement in process efficiency.

### Next‐generation photobioreactor development

The scale up of microalgae systems from the laboratory to commercial facilities theoretically offers the potential to couple H_2_ fuel production to CO_2_ sequestration (e.g. the residual biomass to biochar) or other co‐products. To establish the viability of a given process, rigorous techno‐economic (TEA) and life cycle analyses (LCA), as well as pilot and demonstration scale trials, are required to confirm positive economic returns and ERoEI, as well as reduced GHG emissions. Achieving these targets will not only involve the engineering of next‐generation cell lines as described above, but the design of photobioreactors able to deliver optimized production conditions for the chosen production strains and specific geographic locations (Franz *et al*., 2012; Slegers, 2014; Slegers *et al*., 2011).

## Other artificial and naturally derived renewable H_2_‐production strategies

To replace fossil fuel‐based H_2_ production processes, several renewable H_2_ production strategies are being explored. In addition to biological systems such as algae, these include biologically based or biologically inspired systems as well as physical systems such as PV, wind and wave power. The ability to use electrolysis at peak electricity production times to generate H_2_ for energy storage is already being employed by current European demonstration projects such as HyUnder, INGRID, MYRTE and GRHYD (AREVA, 2013; D'Errico and Screnci, 2012; Darras *et al*., 2012; ENGIE; HyUnder, 2012; INGRID; Poggi *et al*., 2014). Coupling such energy capture and electrolysis processes is more environmentally friendly than using fossil fuels, and could technically provide workable solutions which can reportedly achieve photon‐to‐electron conversion efficiencies of 20% (Bonke *et al*., 2015). Nevertheless, challenges to the scale up of these technologies remain. These include the following: (i) driving down the capital costs of production, (ii) eliminating rare earth metals (Kapdan and Kargi, 2006), (iii) increasing the catalyst longevity and (iv) improving the ERoEI towards a value of 10:1 required to support a civilized society (Hall *et al*., 2009).

Microalgae systems currently only have photon conversion efficiencies of 3% (Scoma *et al*., 2012; Volgusheva *et al*., 2013) and a theoretical maximum of ~12–14% (Melis, 2009; calculated based on the Z‐scheme of electron transport and mean energy of PAR), which is lower than that of artificial systems (Bonke *et al*., 2015). However, they are self‐replicating and repairing, have water splitting complexes and hydrogenases based on manganese, calcium and iron (not rare Earth metals) and have been proven to be able to support H_2_ production over long periods (Laurinavichene *et al*., 2006). Furthermore, H_2_‐producing microalgae can be used in co‐production strategies which theoretically have the potential to improve economic performance (Stephens *et al*., 2010a). More detailed TEA and LCA are required to establish the pros and cons of each technology and which system is more competitive economically in terms of ERoEI and GHG emissions.

Biological H_2_ production can, apart from the above discussed oxygenic photosynthesis, further be achieved via microbial electrolysis cells, dark fermentation and photo‐fermentation (nonoxygenic photosynthesis) (Chandrasekhar *et al*., 2015).

### Microbial electrolysis cells

are designed to produce H_2_ from electrogenic micro‐organisms which consume and release e^−^, H^+^ and CO_2_. An externally supplied voltage allows the formation of H_2_ gas from the released H^+^. As the H_2_ production process itself does not occur inside the cell, microbial electrolysis cells are strictly speaking not bio‐H_2_ production platforms. Additionally, due to the requirement of the external supply of an electric current and carbon‐based feed stocks, the economic viability of scaling up this H_2_ production strategy must be carefully considered (Heidrich *et al*., 2014).

### Dark fermentation

involves the production of H_2_ using facultative or obligate anaerobic bacteria which also consume organic material. However, this is a chemical energy conversion process rather than a renewable energy process. Furthermore, while the process occurs at a higher rate compared to photo‐fermentation or photolysis, it has a low H_2_ yield: substrate use ratio, mainly because of the formation of various volatile by‐products including ethanol, acetic acid, propionic acid and butyric acid (Jones *et al*., 2015), from which H_2_ must be purified (Chandrasekhar *et al*., 2015). Production factors such as tight control of the pH, carbon/nitrogen ratio of the substrate and high temperature requirements add additional challenges and operating costs (Jones *et al*., 2015; Wang *et al*., 2012).

### Anoxygenic photo‐fermentation

is performed by purple nonsulphur photosynthetic bacteria under anaerobic and anoxic conditions. These organisms use solar energy to convert organic compounds such as organic acids, sugars and glycerol into H_2_ and CO_2_. While O_2_ sensitivity of the key enzyme, nitrogenase, is not of concern as these bacteria conduct nonoxygenic photosynthesis, the conversion of organic substrates to H_2_ appears unlikely to be economical or scalable and the abstraction of these substrates (e.g. sugars) from the food chain is undesirable. Furthermore, H_2_ production occurs in the absence of NH_4_ due to the high sensitivity of the nitrogenase to nitrogen in the medium. The main challenges of this process are bacterial contamination, substrate type and carbon/nitrogen ratio, synthesis of competing by‐products such as polyhydroxybutyrate, sensitivity to temperature and light fluctuations, the low catalytic activity of the nitrogenase, expression suppression by NH_4_ (and thus required dilution of waste as a substrate) and lower photochemical efficacy, which collectively lead to low H_2_ production levels (Chandrasekhar *et al*., 2015; Hay *et al*., 2013).

The bio‐H_2_ production routes described above have the advantage of being able to utilize carbon sources which would otherwise be lost, for example from waste water, while assisting in waste water management. However, these processes can still be regarded as chemical energy conversion technologies and are not strictly speaking net solar fuel technologies in which the energy from light is captured and stored in the form of fuel.

## Microalgae H_2_ systems within a global context

It is also important to understand the global context into which this technology fits, as hydrogen sits at the nexus of several global challenges. In 2010, the G20 nations accounted for 83% of global energy demand and similar levels of CO_2_ emissions (BP, 2013). The highest *national* CO_2_ emissions levels were reported in China (8 205.9 Mt CO_2_), the US (5 074.1 Mt CO_2_) and India (1 954.0 Mt CO_2_) (IEA, 2014). *Per capita*, CO_2_ emissions of each of these G20 nations (2013) range from the highest in Saudi Arabia (~17.2 tCO_2_/year/person) to the lowest in the high ‘national emission country’ India (1.8 tCO_2_/ year/person) (GCP, 2013; PRB, 2013). Not surprisingly, this has resulted in a debate between high (e.g. the US, Saudi Arabia and Australia) and low (such as China and India) *per capita* CO_2_ emitters, all of whom are seeking to limit their emission liabilities. Hydrogen as stored chemical energy offers the potential to provide the required *per capita* energy demand without the CO_2_ emissions associated with carbon‐based fuels. It also offers significant advantages in terms of the development of global distributed systems and networks, as it can be produced using a broad range of technologies making it an important energy currency. However, significant challenges remain. Some of the greatest include market penetration given the dominance of fossil fuels, the high cost of replacing existing carbon‐based fuel infrastructure and the technical difficulty of capturing, storing, purifying and compressing H_2_ (Bimbo *et al*., 2013; Hruzewicz‐Kołodziejczyk *et al*., 2012).

In terms of the photosynthetic H_2_ production component of this infrastructure, considerable lessons can be learnt from demonstration scale systems for nearer market volatile fuels, for example ethanol, which are reportedly close to commercial production using cyanobacteria for less than $2 per gallon (Perkins, 2014). Insights can be gained in terms of capital costs and their potential reduction, strategies to optimize light delivery, as well as methods to capture volatile products as part of a strategy to minimize feedback inhibition.

In terms of H_2_ storage and distribution systems, there is as yet no global infrastructure enabling the use of H_2_ as a ‘drop‐in’ fuel. Nevertheless, H_2_ is widely used in industry and this provides a solid knowledge base to build on (Ramachandran and Menon, 1998). Additionally, during the early stages of deployment, storage and distribution are not critical for the productive use of H_2,_ as H_2_ can be used to reduce CO_2_ to yield a range of CO_2_ neutral carbon‐based drop‐in fuels (Centi and Perathoner, 2009; Wang *et al*., 2011) compatible with existing storage and distribution systems. Notably, forecasts indicate that by 2025, H_2_ may contribute 8–10% to the energy market (Gupta *et al*., 2013), which provides a development driver for H_2_ production, storage, distribution and conversion technologies, and these are actively being developed and deployed internationally (see below). Policies such as tax incentives are also gradually being established (FCHEA, 2014). Consequently, an international movement is beginning to connect solar H_2_‐producing technologies with advancements in storage, distribution and fuel cell technologies.

## The patent landscape

**Figure 2:**
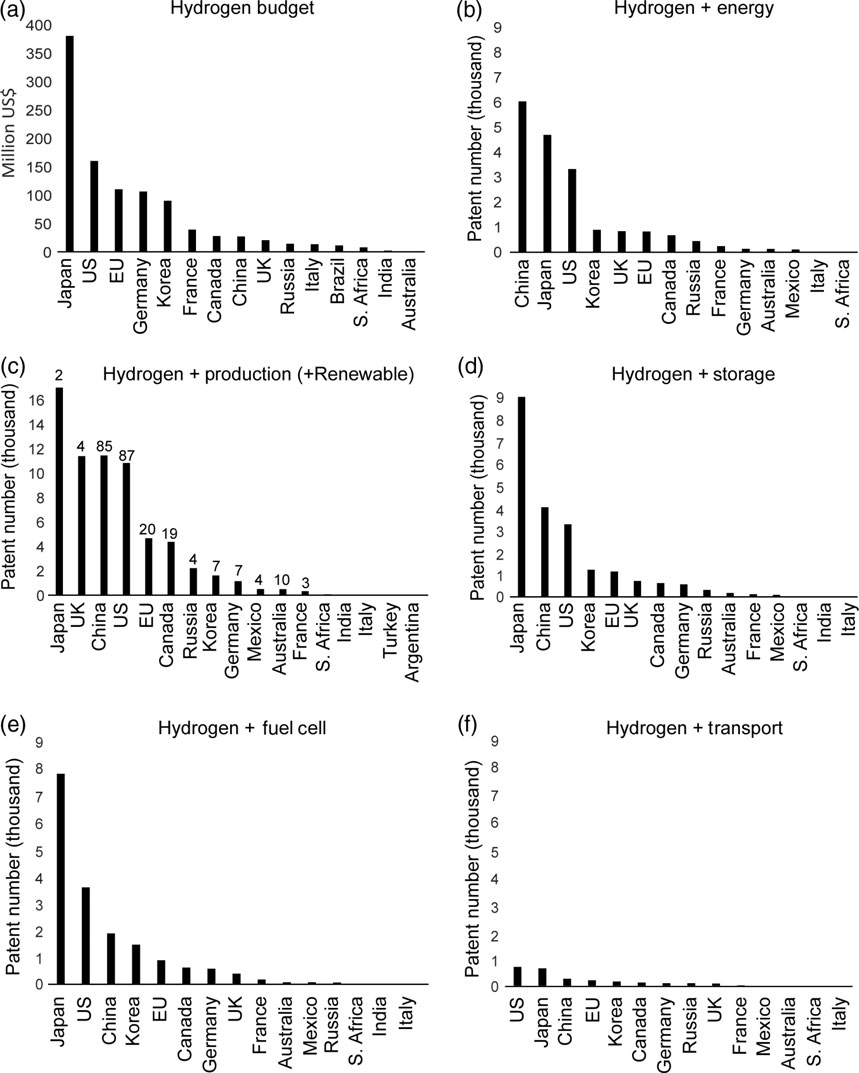
Hydrogen budget and potential market in G20 countries. (a): Allocated budget per country for the development of hydrogen technology. (b–f) Patents filed in the respective countries. Numbers derived from the European Espacenet world wide database keyword search using: Hydrogen + Energy, Hydrogen + Production and Hydrogen  +  Production  +  Renewable (renewables are shown as absolute patent numbers above bar), Hydrogen + Storage, Hydrogen + Fuel cell, Hydrogen + Transport.

Analysis of the global H_2_ patent landscape provides valuable insights for a developing microalgal H_2_ biotechnology industry, in terms of both challenges and opportunities. National expenditure levels on H_2_ technologies by the G20 nations are shown in Figure 2a, with Japan, the US and the EU supporting the largest number of developments. Current patent submissions in the G20 countries in the areas of H_2_ energy (Figure 2b) and H_2_ production (Figure 2c), as well as renewable H_2_ production (Figure 1c, absolute patent numbers given above bar), H_2_ storage (Figure 2d), H_2_ fuel cells (Figure 2e) and H_2_‐driven transport (Figure 2f), highlight the advances in the delivery of these components of a future H_2_ economy.

The majority of patents related to H_2_ as a renewable energy source were filed in the US and China (Figure 2c; 87 and 85 patents, respectively), countries which are among the highest *national* CO_2_ emitters. Japan reported the highest H_2_ fuel cell patent submissions (Figure 2e), consistent with its stated aim of transitioning to a H_2_ fuel future (GoJ, 2014). These international patenting patterns may be an indicator of early technology adopter hotspots and highlight the potential of developing renewable H_2_ production platforms to supply emerging global H_2_ markets and distribution networks.

## Conclusions

Solar‐driven microalgal H_2_ production is both a promising and challenging biotechnology, and will likely play an important role in the global drive to reduce GHG emissions. Advances in molecular engineering, TEA/LCA and pilot scale test facilities and predictive design are providing critical tools for the development of the solar fuel systems required to tap into the huge solar resource available to us and which could be used to drive a sustainable energy economy. Using these tools to develop ‘deployment ready’ microalgae or artificial solar fuel systems in the next 10 years is of critical importance for climate stabilization, but this can only be achieved within a framework of political leadership which will enable up to 70% emission reductions within the next 10–20 year time window.
